# The impact of age, sex, and comorbidities on COVID-19 mortality of hospitalized patients during the SARS-CoV-2 pandemic: data from the multicentric prospective cohort study of the Lean European Open Survey on SARS-CoV-2 (LEOSS)

**DOI:** 10.1007/s15010-025-02583-z

**Published:** 2025-06-17

**Authors:** Julian Triebelhorn, Maria M. Rüthrich, Susana M. Nunes de Miranda, Jochen Schneider, Timm Westhoff, Margarete Scherer, Christoph D. Spinner, Maria J. G. T. Vehreschild, Florian Voit, Julia Lanznaster, Johanna Erber, Kerstin Hellwig, Bjoern-Erik Ole Jensen, Laura Wagner

**Affiliations:** 1 Department of Internal Medicine II, Technical University of Munich, TUM School of Medicine and Health, TUM University Hospital, Munich, Germany; 2 Department of Nephrology and Medical Intensive Care, Charité Berlin University Medicine, Berlin, Germany; 3 Department of Internal Medicine II, Hematology and Medical Oncology, University Hospital Jena, Jena, Germany; 4 Faculty of Medicine and University Hospital Cologne, Department I for Internal Medicine, University of Cologne, Cologne, Germany; 5 Department of Internal Medicine I, Marien Hospital Herne Ruhr University Bochum, Herne, Germany; 6 Faculty of Medicine, Goethe University Frankfurt, Institute for Digital Medicine and Clinical Data Science, Frankfurt Am Main, Germany; 7 Department of Internal Medicine, Infectious Diseases, University Hospital Frankfurt Goethe University Frankfurt, Frankfurt Am Main, Germany; 8 Department of Internal Medicine II, Hospital Passau, Passau, Germany; 9 Department of Neurology, St. Josef-Hospital Bochum, Ruhr University Bochum, Bochum, Germany; 10 Department of Gastroenterology, Hepatology and Infectious Diseases, Medical Faculty and University Hospital Düsseldorf, Heinrich Heine University, Düsseldorf, Germany

**Keywords:** COVID-19, SARS-CoV-2, Pandemic, Mortality, Vaccination, Variant

## Abstract

**Purpose:**

This study aimed to analyse COVID-19-related mortality during the pandemic, stratified by groups at risk of severe COVID-19.

**Methods:**

Patients with COVID-19 between March 2020 and February 2023 were enrolled using the international multicentric Lean European Open Survey on SARS-CoV-2-Infected Patients (LEOSS). The COVID-19 in-hospital mortality was calculated using a multivariable logistic regression model adjusted for age and sex.

**Results:**

A total of 11,765 patients were included, with an overall mortality rate of 13.1% (N = 1541). Mortality decreased from 14.4% during the wildtype (wt) period to 10.6%, 9.5%, and 6.3% in the alpha (α), delta (δ), and omicron (Ω) periods, respectively. Patients aged 66–75, 76–85, and > 85 years had 11.4-, 19.3-, and 34.7-fold higher mortality odds than patients aged 26–35 years (p < 0.001 in all comparisons). This increase in mortality between younger and older patients decreased with the shift from wt (increase of 39.4%) to Ω (15.5%). The overall adjusted mortality rate in males (18.4%) was higher than in females (10.6%); however, this sex-specific difference levelled off with the shift from wt (m: 18.9%, f: 10.1%) to Ω (m: 5.9%, f: 5.3%). Referring to comorbidities, adjusted mortality increased significantly with the number of comorbidities in patients during the wt but remained stable in patients with Ω-period. Among severely immunosuppressed patients, mortality declined markedly throughout the pandemic (wt vs. Ω: p < 0.001).

**Conclusion:**

Overall mortality decreased during the pandemic, even among severely immunosuppressed patients. Age, sex, and the number of comorbidities were key mortality risk factors, although their impact lessened as the pandemic progressed.

**Supplementary Information:**

The online version contains supplementary material available at 10.1007/s15010-025-02583-z.

## Introduction

The COVID-19 pandemic has dramatically impacted global public health, causing significant morbidity and mortality during the initial waves. Throughout the pandemic, COVID-19 mortality has decreased, driven by the emergence of less lethal variants, increasing population immunity, and advancements in treatment options [1].

Previous studies have identified that older patients, particularly those aged 65 years and above, are at the highest risk of severe disease due to frailty, comorbidities, and immune senescence [2]. Furthermore, male sex and a higher number of comorbidities are associated with severe disease [3–5]. Patients with comorbidities such as immunosuppression, cancer, chronic cardiac, pulmonary, and kidney diseases, obesity, and chronic neurological disorders are at an increased risk of hospitalisation and death from COVID-19 [2, 4, 6, 7]. Immunosuppression encompasses a heterogeneous spectrum: Agrawal et al. found that patients who had undergone solid organ, renal, or bone marrow transplantation faced the highest mortality risk [2], while Turtle et al. reported elevated mortality rates among patients with active cancer or those receiving steroids [6].

The changing significance of these common risk factors throughout the pandemic, particularly during the Omicron era, has not been thoroughly explored. Although treatment options have improved, Turtle et al. observed that in-hospital mortality from COVID-19 decreased between the Wildtype (wt) and Omicron (Ω) variants for most patients but remained disproportionately higher for immunosuppressed individuals [6]. However, the variant-specific impact of age, sex, and comorbidities as risk factors for severe COVID-19 warrants further investigation.

This study aimed to analyse COVID-19 mortality throughout the pandemic, focusing on the influence of key risk factors, including age, sex, and comorbidities. It seeks to identify patient groups that have particularly benefited from variant shifts, and enhanced treatment strategies.

## Methods

### Data collection and study population

Data were obtained from the Lean European Open Survey on SARS-CoV-2 infected patients (LEOSS) registry, a multi-centre non-interventional cohort study. The LEOSS registry included outpatient and hospitalized patients with confirmed SARS-CoV-2 infection verified by polymerase chain reaction (PCR) and nosocomial COVID-19 from 136 study sites in 11 European countries. Most patients (96.6%) were enrolled in Germany, followed by Turkey (1.1%), with other countries contributing 2.3% collectively (Belgium (0.6%), Latvia (0.3%), Czech Republic (0.3%), Italy (0.2%), Great Britain (0.2%), and others (0.7%)). Clinical data were collected anonymously using an electronic case report form. Among 13,272 patients in the LEOSS cohort, this study included data from patients hospitalized with laboratory-confirmed SARS-CoV-2 infection between March 2020 and February 2023. Patients in palliative care, asymptomatic, outpatients, and patients aged < 26 years were excluded. The reason for the exclusion of patients aged < 26 years was the small number of cases, particularly during the omicron period, and the high amount of missing data regarding mortality in this age group.

### Variables

#### SARS-CoV-2 periods

PCR-confirmed SARS-CoV-2 infections were categorised based on infection date and epidemiological data regarding dominating SARS-CoV-2 variant from the Robert Koch Institute in Germany [8]. All SARS-COV-2 infections between 01/2020 and 01/2021 were considered as occurred during the wt, between 02/2021 and 06/2021 alpha (α)-, between 07/2021 and 12/2021 delta (δ)-, and between 01/2022 and 02/2023 Ω-period.

#### Age groups

Age-outcome relationships were calculated for the following groups: 26–35, 36–45, 46–55, 56–65, 66–75, 76–85, and > 85 years.

#### Comorbidities

Comorbidities were aggregated into groups due to low incidence rates for some comorbidities, particularly in the later periods. Seven comorbidity groups were created: cardiovascular disease (myocardial infarction, chronic heart failure, peripheral vascular disease, hypertension, atrial fibrillation, coronary artery disease), pulmonary disease (chronic obstructive pulmonary disease, asthma, other chronic pulmonary diseases), renal disease (acute and chronic kidney disease), liver disease (chronic liver disease, liver cirrhosis), cancer (metastatic and non-metastatic), severe immunosuppression (leukaemia, lymphoma, stem cell transplantation, solid organ transplantation, human immunodeficiency virus, autoimmune disease, immunosuppressive medication (corticosteroids, methotrexate, azathioprine, ciclosporin, calcineurin-inhibitors, mTOR-inhibitors)), diabetes (type 1 and type 2 diabetes). 

#### Vaccination

Patients who had received at least 1 dose of any authorised COVID-19 vaccine were classified as vaccinated.

### Outcomes

The primary outcomes were the observed in-hospital mortality, and the adjusted mortality, determined in a multivariable regression model.

### Statistical analysis

Patient characteristics for categorical variables were expressed as absolute numbers and percentages. The probability of in-hospital mortality across pandemic phases was modelled using univariate and multivariable logistic regression. Univariate analysis provided odds ratio (OR) and 95% confidence interval (CI) estimates, while multivariable models adjusted for age and sex. Interaction effects between the period of SARS-CoV-2 period and sex or comorbidity indicators were tested to derive period-specific comorbidity effects.

Observed mortality outcomes were analysed alongside adjusted mortality calculated from multivariable regression models. Observed mortality refers to the crude proportion of in-hospital deaths in the study cohort. Adjusted mortality refers to the rate estimated by a multivariable model that controls for the confounders age and sex. Adjusted mortality was reported as mean probability with 95% confidence intervals (CIs). Changes in adjusted mortality were assessed by comparing patients with wt to those with Ω, reporting OR and p-values. All reported p-values were calculated 2-sided, p < 0.05 was considered statistically significant. All statistical analyses were performed using R version 4.4.2 (R Foundation for Statistical Computing, Vienna, Austria). Adjusted probabilities of COVID-19-related death and multiple comparisons were calculated using the marginal effects package [9]. Multiple imputation was used to account for missing data using the mice package [10]. Predictive Mean Matching (PMM) was used on missing data in comorbidity groupings.

The number of imputations was set to 10. Model estimates, or quantities of interests in multiple comparisons respectively, were pooled using Rubin’s rule.

## Results

### Study cohort

**Fig. 1:**
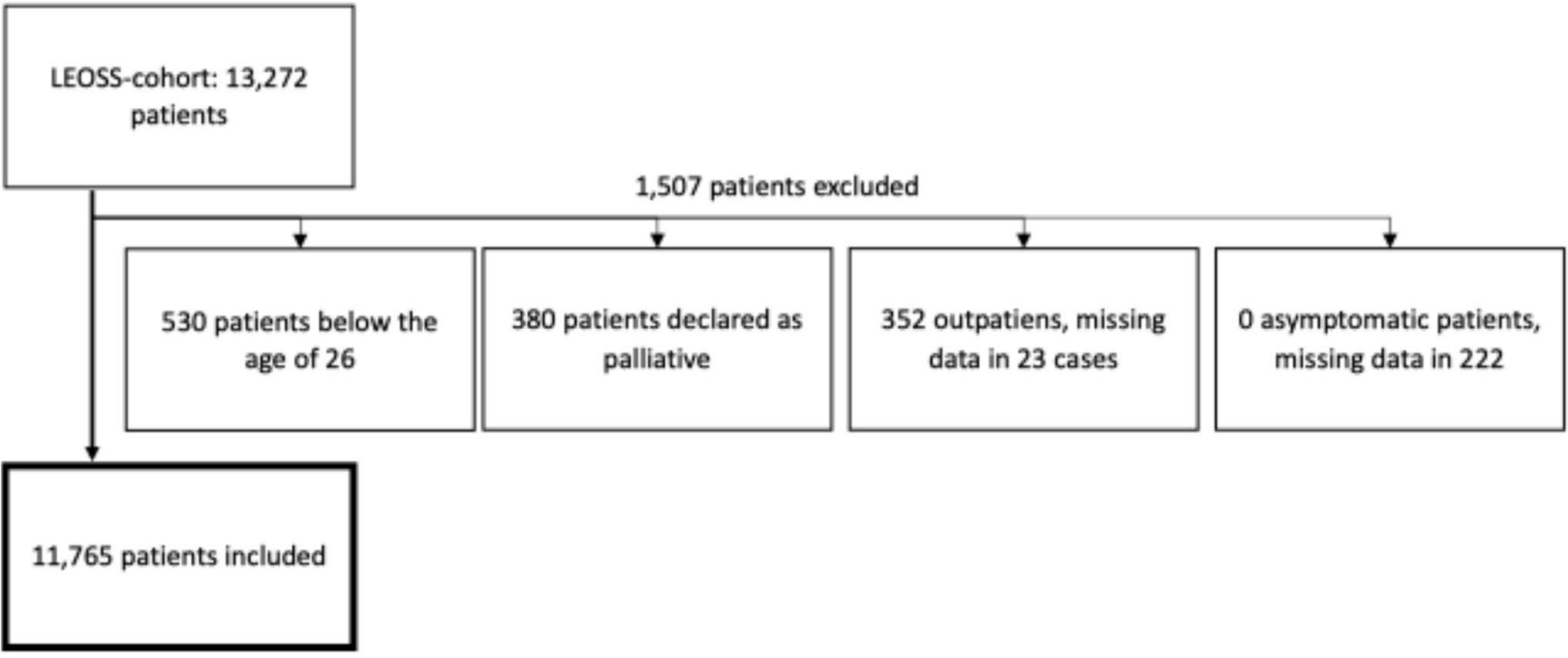
Overview of the study cohort

The dataset included 11,765 patients hospitalized with laboratory-confirmed SARS-CoV-2 infection. After exclusion of 380 patients in palliative care, 352 outpatients, and 530 patients aged < 26 years. There were no asymptomatic patients (Fig. 1). The median age group was 66–75 years. In terms of ethnicity, 9,043 patients (76.9%) were of Caucasian origin, followed by 2311 patients (19.6%) of unknown origin, 278 patients (2.4%) identified as Asian or Pacific Islander, and 104 patients (0.9%) identified as African or African American. Overall, 2604 patients (21.6%) required intensive care unit (ICU) treatment, and 1541 patients (13.1%) died in hospital.

### Observed mortality rate in relation to SARS-CoV-2 period

Of the 11,765 hospitalized patients, 8800 (74.8%), 1277 (10.9%), 897 (7.6%), and 791 (6.7%) were infected during the wt-, α-, δ-, and Ω period, respectively. Patients infection during the wt period had the highest observed mortality rate (14.4%), followed by the α- (10.6%), δ- (9.5%), and Ω period (6.3%). The decrease in observed mortality was statistically significant (OR Ω vs. wt = 0.4; p < 0.001) (Table 1, see web-only Supplementary Figure S1).

**Table 1:** Observed mortality rate depending on SARS-CoV-2 period

SARS-CoV-2 period	Total (N = 11,765)	No Death (N = 10,244)	Death (N = 1,541)	Mortality rate (%)
Wildtype	8800 (74.8%)	7530 (73.5%)	1270 (82.4%)	14.4%
Alpha	1277 (10.9%)	1141 (11.1%)	136 (8.8%)	10.6%
Delta	897 (7.6%)	812 (7.9%)	85 (5.5%)	9.5%
Omicron	791 (6.7%)	741 (7.2%)	50 (3.2%)	6.3%

*No* number

### Adjusted mortality in relation to age and SARS-CoV-2 period

**Fig. 2:**
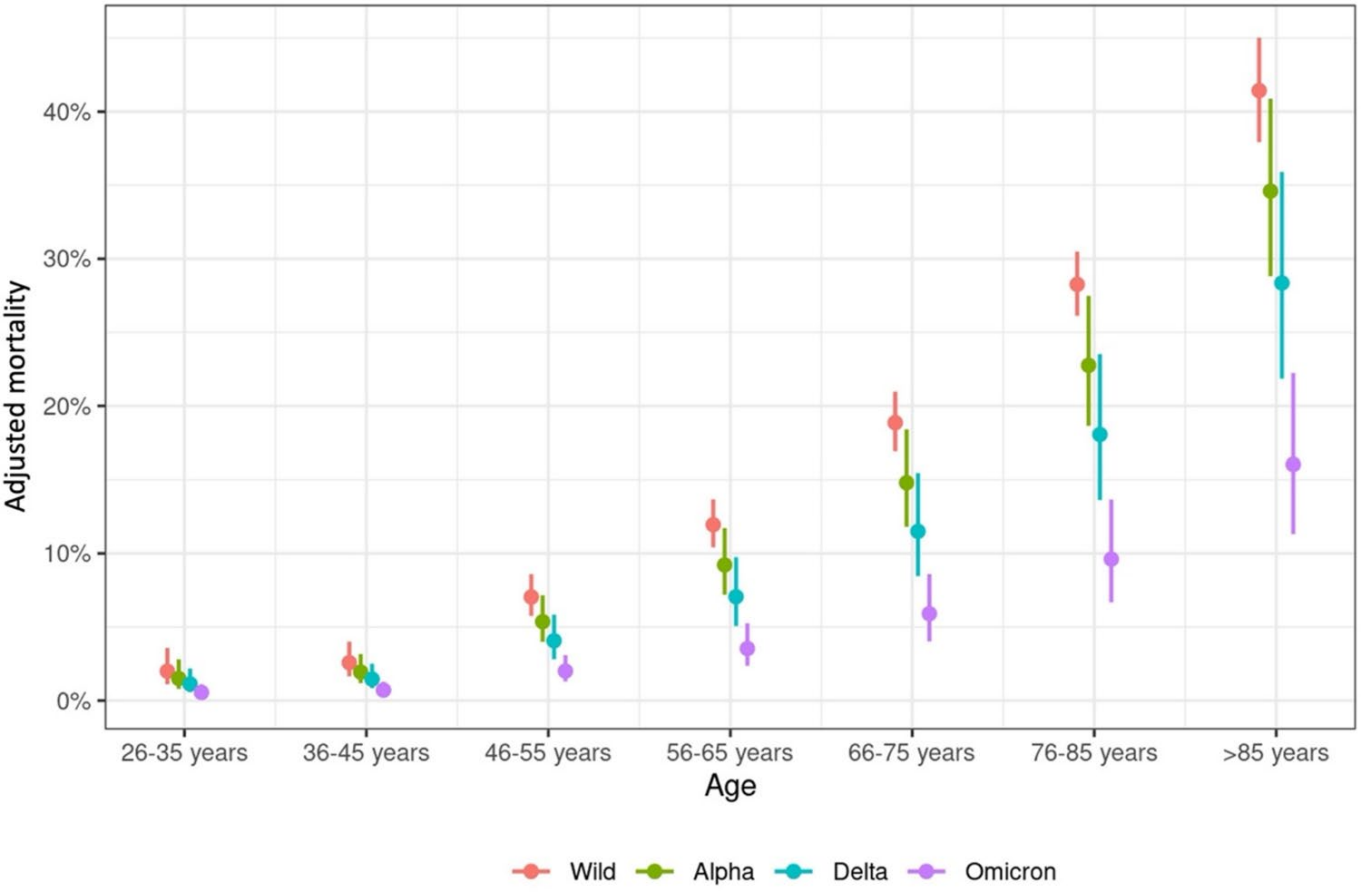
Adjusted mortality rate in relation to age and SARS-CoV-2 period

Among the participants, 822, 1056, 1716, 2160, 2117, 2705, and 1189 were aged 26–35, 36–45, 46–55, 56–65, 66–75, 76–85, and > 85 years, respectively (See web-only Supplementary Table S1). Across all periods of SARS-CoV-2 periods, age significantly influenced mortality. Patients aged 66–75, 76–85, and > 85 years had 11.4-, 19.3-, and 34.7-fold higher mortality odds than those aged 26–35 years (p < 0.001 in all listed comparisons). The adjusted mortality rate increased with age, with patients > 85 years having the highest adjusted mortality rate of 40.7% across all SARS-CoV-2 periods (See web-only Supplementary Table S1). The correlation between age and probability of COVID-19-related death persisted across all SARS-CoV-2 periods, although it decreased overall during the pandemic (Fig. 2). The difference in adjusted mortality between younger (age 26-–35 years, adjusted mortality: wt 2%, Ω 0.5%) and older patients (age > 85 years; adjusted mortality: wt 41.4%, Ω 16.0%) decreased with the shift from wt- (increase of 39.4%) to Ω period (increase of 15.5%) (see web-only Supplementary Table S2).

### Adjusted mortality in relation to sex and SARS-CoV-2 period

**Fig. 3:**
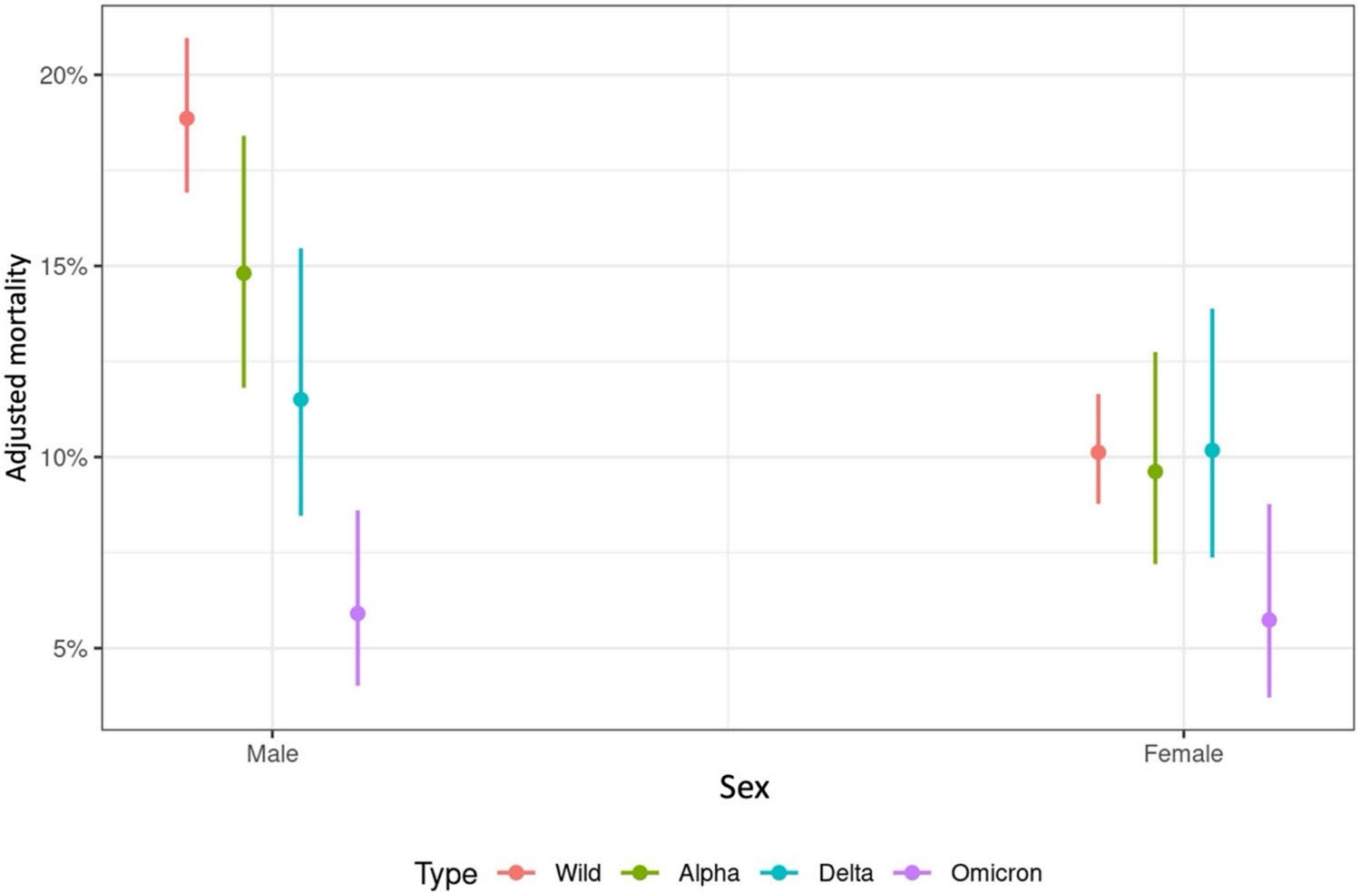
Adjusted mortality rate in relation to sex and SARS-CoV-2 period

Among the 11,765 patients, 6687 (56.8%) were male and 5078 (43.2%) were female. Adjusted mortality significantly decreased for both sexes when comparing wt and Ω period (male: OR = 3.7, p < 0.001; female: OR = 1.8, p = 0.008). Adjusted mortality rates were consistently lower in females (10.6%) than in males (18.4%) (OR = 0.48; p < 0.001) (see web-only Supplementary Table S3). Analysing the adjusted probability of COVID-19-related death by sex and SARS-CoV-2 period, the mortality rate for male patients progressively approached that of female patients throughout the pandemic (Fig. 3). In the multivariable logistic regression model adjusted for patients aged 66–75 years, adjusted mortality significantly differed between males and females during the wt- (p < 0.001) and α-period (p = 0.011) but not during the δ- (p = 0.561) and Ω-period (p = 0.917). This difference in mortality rates between sexes during the δ and Ω-periods was consistent across all age groups. (See web-only Supplementary Figure S2 and Table S4).

### Adjusted mortality in relation to comorbidities throughout the pandemic

**Fig. 4:**
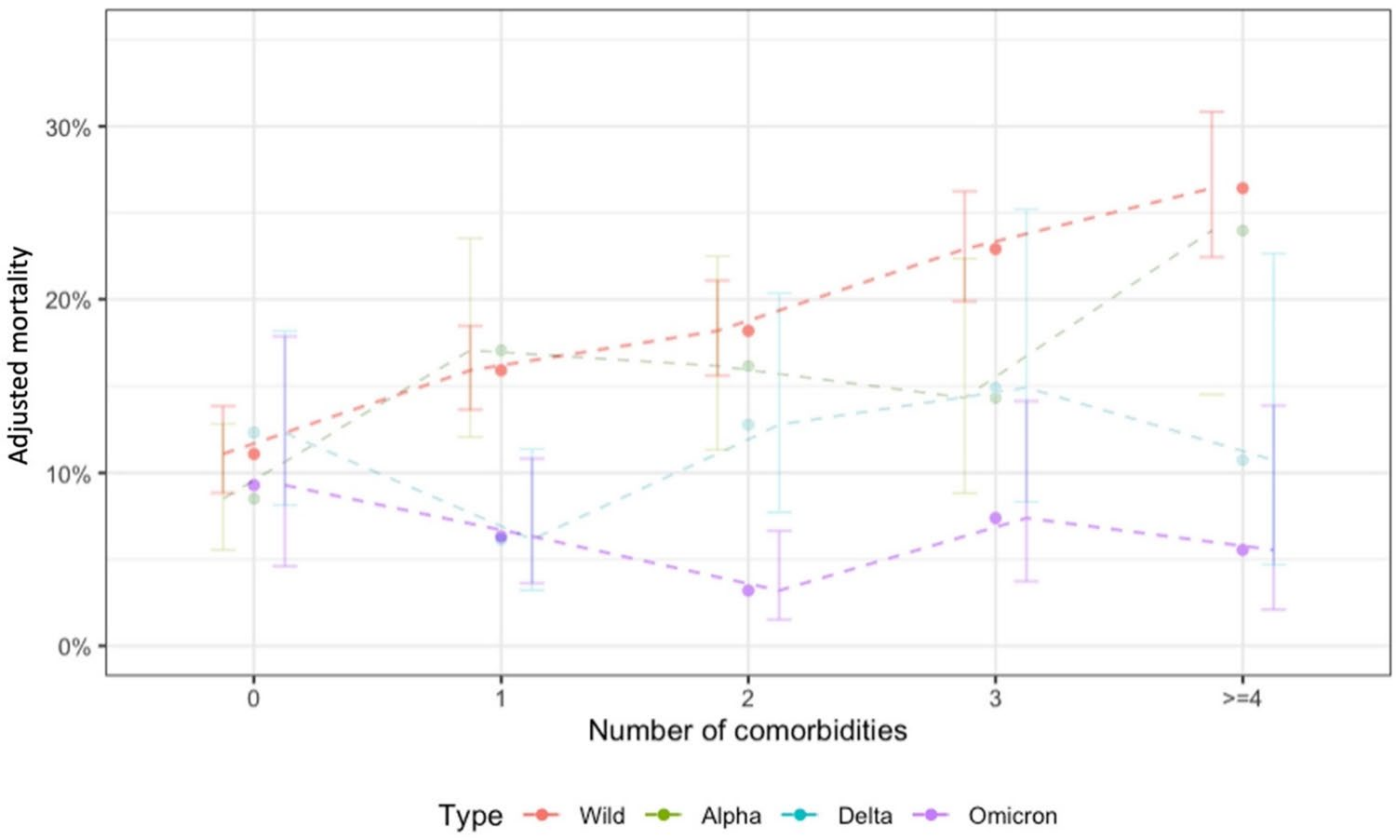
Adjusted mortality rate in relation to SARS-CoV-2 period and number of comorbidities

The number of comorbidities significantly impacted mortality during the wt-period compared to the Ω-period. Patients with 1 to >  = 4 comorbidities infected with wt had higher adjusted mortality than those with the same comorbidities infected with Ω (p < 0.001 for all comparisons, see web-only Supplementary Table S5, Fig. 4). No significant difference was observed in adjusted mortality between patients with no comorbidities comparing wt- and Ω-period (p = 0.61). The adjusted mortality rate of patients infected during the Ω period was independent of the number of comorbidities (comparison of 0 vs. ≥ 4 comorbidities, p = 0.424). Patients infected during the wt-period had a higher adjusted mortality rate, as more comorbidities were present (comparison of 0 vs. ≥ 4 comorbidities, p < 0.001) (see web-only Supplementary Table S6). Patients with cardiovascular disease (wt vs. Ω: OR = 3.36, p < 0.001) and severe immunosuppression (wt vs. Ω: OR = 7.39, p < 0.001) exhibited significant declines in mortality during the Ω period. No other comorbidity groups showed significant changes in mortality comparing wt- with Ω period (see web-only Supplementary Table S7, and Figure S3).

### Mortality of the entire cohort in relation to vaccination status and SARS-CoV-2 period

Vaccination rates increased from 0.4% during the wt period to 57.5% during the Ω period (See web-only Supplementary Table S9). Regarding patients that received at least one dose of vaccination, the adjusted mortality showed no significant differences between infection during the wt (p = 0.772), α (p = 0.129), and δ (p = 0.078) period. However, unvaccinated patients infected during Ω period had a higher adjusted mortality rate compared to patients that received at least one dose of vaccination (p < 0.001) (see web-only Supplementary Table S10, and Figure S4).

### Use of COVID-19-specific antiviral therapy throughout the pandemic

The percentage of patients who received specific COVID-19 therapy with monoclonal antibodies or remdesivir increased over time. This rise was most pronounced in patients with severe immunosuppression. During the Ω period, 152 (60.3%) severely immunosuppressed patients received COVID-19-specific antiviral treatment, compared to 109 (20.9%) non-severely immunosuppressed patients (see web-only Supplementary Table S11, and Figure S5).

## Discussion

COVID-19 mortality decreased significantly throughout the pandemic [1] and it is well known that patients with older age, certain comorbidities, especially immunosuppression, and male sex have a higher mortality risk from COVID-19 [2, 3, 11–13]. The novelty of this study is that it extends these findings by demonstrating how the relative importance of risk factors evolved across the periods within a hospitalized cohort.

This data showed that although adjusted mortality increased with age, this increase was far less pronounced during the Ω period than the wt period, resulting in significantly lower adjusted mortality rates among older adults. Similarly, the sex-specific mortality gap observed during the wt period narrowed progressively with each subsequent variant, nearly disappearing by the Ω period. Peckham et al. [14] reported a similar finding in a meta-analysis conducted in early 2020, demonstrating that male sex was associated with a higher risk of ICU admission and death compared to females. In contrast, during the omicron period, Kopp et al. [15] observed no significant sex differences in cardiovascular (CV) mortality. Notably, however, their study found that men had the highest 18-month CV mortality during the delta period – a result that contradicts our findings. Consistent with our observations, Ward et al. [16] reported a lower mortality risk during the omicron period compared to delta, with a more pronounced risk reduction in males than in females.

The LEOSS registry maintained a nearly equal distribution of male and female patients across all periods, and the convergence of the observed sex-based differences in COVID-19 mortality is unlikely to be explained by changes in sex composition. Instead, it may be attributable to distinct immune responses to SARS-CoV-2 infection in males and females [14, 17, 18]. Males exhibit higher pro-inflammatory chemokines and cytokines levels, and females show stronger T cell-mediated responses [19]. These immunological distinctions likely contribute to more efficient viral control and a reduced risk of severe disease progression in females. The narrowing of the sex-specific mortality gap over the course of the pandemic could be attributed to the demonstrably reduced virulence of omicron [20]. In addition, an effect from increasing immunity through infections and vaccinations, and improved treatment options, is to be assumed.

Regarding multimorbidity, the association between the number of comorbidities and increased mortality observed during the wt period was not present during the Ω period. Nyberg et al. [21] found a lower risk of hospital admission, admission to ICU, and death for patients infected with Ω compared to δ. The difference was especially pronounced in unvaccinated and booster-vaccinated patients and less in patients who received only one or two vaccine doses. Similarly, the COVID-19 Omicron Delta study group [22] found a 40% higher 30- and 60-day survival in hospitalized patient with COVID-19 and Omicron compared to Delta, especially for patients who had received three vaccine doses. One of the main findings in our study was that the in-hospital mortality declined from wt- to Ω period. We found a decline in in-hospital mortality both in vaccinated (at least one dose) and unvaccinated patients, but the decline was more pronounced in vaccinated patients. Unexpectedly, no difference in in-hospital mortality could be found comparing vaccinated patients infected during the α- and δ period, which could be due to a high amount of missing data regarding vaccination status. In this study, severely immunosuppressed patients and those with cardiovascular disease demonstrated a significant decline of in hospital-mortality throughout the pandemic. Given that vaccinations have been found to significantly decrease cardiovascular events and complications during COVID-19, patients with cardiovascular illnesses may have benefited most from them [23, 24]. Notably, the decline of relative risk was most pronounced among severely immunosuppressed patients, with an OR of 7.4 in the adjusted analysis, indicating substantial risk reduction. Similar observations were published by Turtle et al. (6), describing a relevant decline in mortality for immunocompromised patients from 36% during wt period to 19% during the Ω period. Overvad et al. [25] found a higher risk of hospital contact with COVID-19, severe COVID-19, and hospitalization followed by death in solid organ transplant recipients. Regarding Ω, the risk of hospitalization and death after SARS-CoV-2 infection declined in their study, which supports the findings of our study. The disproportional use of COVID-19-specific therapies in immunosuppressed patients, which have been shown to significantly lower COVID-19-related death [26], particularly during the early infection phase, likely contributed to this substantial decline of risk in immunosuppressed patients.

By further investigating the overall decline in mortality among hospitalized COVID-19 patients concerning defined risk factors, this study identified that older, male, and immunosuppressed patients disproportionately benefited from changes in variants and COVID-19-specific therapies. Future research should aim to better define subgroups, such as patients with specific immune deficiencies, who may still derive significant benefit from targeted antiviral therapies, even when overall mortality declines. The effect of vaccination could not be definitively investigated in this study, but other studies should be mentioned that show that vaccination still reduces both the risk of infection and disease by SARS-CoV-2, especially with variant-adapted vaccines [27, 28].

This study has several limitations. The findings are not generalisable to the broader population, as the analysis was limited to hospitalized patients. Additionally, the data was unevenly distributed, with a significantly higher number of patients infected during the wt than α, δ, or Ω period, potentially biasing period-specific analyses. This disparity is likely due to reduced efforts to include hospitalized patients with SARS-CoV-2 and fewer hospitalisations as the pandemic progressed. No genotypic studies were carried out and the data on the SARS-CoV-2 variants relate exclusively to the respective period of infection. Due to the inherently skewed distribution of vaccination data—characterized by low vaccination rates in wt and α, with predominantly single-dose administration, and substantial missing data, a comparative analysis was conducted between patients who had received at least one vaccine dose and those who were unvaccinated. This may have affected the measured impact of vaccination. Hansen et al. [29] reported that a previous infection with Ω provided protection against BA.5 and B.2 infection and hospitalization in triple-vaccinated individuals. This important aspect could not be targeted in this study due to substantial missing data regarding previous SARS-CoV-2 infections. Another limitation is the lack of detailed information on immunosuppression. Severe immunosuppression was defined solely by the type of medication, with no dosage data available, which may have led to an overestimation of its severity in some cases.

In conclusion, this prospective analysis of the LEOSS registry demonstrated that the influence of traditional risk factors for COVID-19-related mortality, including age, sex, and comorbidities, decreased throughout the pandemic, becoming less prominent during the Ω period. Changes in SARS-CoV-2 variants collectively contributed to a significant reduction in overall COVID-19-related mortality. The decline was disproportionally notable among severely immunosuppressed patients and those with cardiovascular diseases, highlighting the benefits of targeted interventions in the most vulnerable groups during the later stages of the pandemic. 

## Supplementary Information

Below is the link to the electronic supplementary material.

Supplementary file1 (DOCX 533 KB)

## Data Availability

No datasets were generated or analysed during the current study.
