# Supplementary material for: The impact of age, sex, and comorbidities on COVID-19 mortality of hospitalized patients during the SARS-CoV-2 pandemic: data from the multicentric prospective cohort study of the Lean European Open Survey on SARS-CoV-2 (LEOSS)
Source: Infection. 2025 Jun 17;53(6):2481–9. doi: 10.1007/s15010-025-02583-z (PMC12675564; doi:10.1007/s15010-025-02583-z)
Supplement: Supplementary file 1 — Supplementary file1 (DOCX 533 KB) [file 15010_2025_2583_MOESM1_ESM.docx]

**Online Supplement to: The impact of age, sex, and comorbidities on COVID-19 mortality of hospitalized patients during the SARS-CoV-2 pandemic: Data from the multicentric prospective cohort study of the Lean European Open Survey on SARS-CoV-2 (LEOSS).**

Julian Triebelhorn^1#^, Maria M. Rüthrich^2,3^, Susana M. Nunes de Miranda^4^, Jochen Schneider^1^, Timm Westhoff^5^, Margarete Scherer^4,6^, Christoph D. Spinner^1^, Maria J.G.T. Vehreschild^7^, Florian Voit^1^, Julia Lanznaster^8^, Johanna Erber^1^, Kerstin Hellwig^9^, Bjoern-Erik Ole Jensen^10^*, and Laura Wagner^1^*

*These authors contributed equally.

^1^ Technical University of Munich, TUM School of Medicine and Health, Department of Internal Medicine II, TUM University Hospital, Munich, Germany

^2^ Department of Nephrology and Medical Intensive Care, Charité Berlin University Medicine, Berlin, Germany

^3^Department of Internal Medicine II, Hematology and Medical Oncology, University Hospital Jena, Jena, Germany

^4^ University of Cologne, Faculty of Medicine and University Hospital Cologne, Department I for Internal Medicine, Cologne, Germany

^5^ Department of Internal Medicine I, Marien Hospital Herne Ruhr University Bochum, Herne, Germany

^6^ Goethe University Frankfurt, Faculty of Medicine, Institute for Digital Medicine and Clinical Data Science, Germany

^7^ Department of Internal Medicine, Infectious Diseases, University Hospital Frankfurt Goethe University Frankfurt, Frankfurt am Main, Germany

^8^ Department of Internal Medicine II, Hospital Passau, Passau, Germany

^9^ Department of Neurology, St. Josef-Hospital Bochum, Ruhr University Bochum, Bochum, Germany

^10^ Department of Gastroenterology, Hepatology and Infectious Diseases, Medical Faculty and University Hospital Düsseldorf, Heinrich Heine University, Düsseldorf, Germany

^#^Corresponding author

Julian Triebelhorn, MD

Technical University of Munich, TUM School of Medicine and Health, Department of Internal Medicine II, TUM University Hospital, Munich, Germany

Email: [julian.triebelhorn@mri.tum.de](mailto:julian.triebelhorn@mri.tum.de)

Fax: 0049 (0) 89 4140 4808

Phone: 0049 (0) 89 4140 9329

Supplementary Tables:

**Supplementary Table S1** Observed mortality rate and adjusted mortality rate in relation to age-group

| Age-group | Total  (N=11,765) | No Death  (N=10,224) | Death  (N=1,541) | Mortality rate, observed | Comparison to patients aged 26–35 years, observed | | Adjusted mortality rate, adjusted to male sex | Comparison to patients aged 26–35 years, adjusted | |
| --- | --- | --- | --- | --- | --- | --- | --- | --- | --- |
|  |  |  |  |  | OR, [CI] | p-value |  | OR | p-value |
| 26–35 years | 822 (7.0%) | 811 (7.9%) | 11 (0.7%) | 1.3% |  |  | 1.9% |  |  |
| 36–45 years | 1,056 (9.0%) | 1,037 (10.1%) | 19 (1.2%) | 1.8% | 1.4 [0.65-2.95] | 0.431 | 2.5% | 1.3 | 0.496 |
| 46–55 years | 1,716 (14.6%) | 1,625 (15.9%) | 91 (5.9%) | 5.3% | 4.1 [2.3-8.22] | 0.001 | 6.9% | 3.7 | <0.001 |
| 56–65 years | 2,160 (18.4%) | 1,965 (19.2%) | 195 (12.7%) | 9.0% | 7.3 [4.16-14.35] | <0.001 | 11.6% | 6.7 | <0.001 |
| 66–75 years | 2,117 (18.0%) | 1,818 (17.8%) | 299 (19.4%) | 14.1% | 12.1 [6.9-23.67] | <0.001 | 18.4% | 11.4 | <0.001 |
| 76–85 years | 2,705 (23.0%) | 2,133 (20.9%) | 572 (37.1%) | 21.1% | 19.8 [11.39-61.03] | <0.001 | 27.7% | 19.3 | <0.001 |
| >85 years | 1,189 (10.1%) | 835 (8.2%) | 354 (23.0%) | 29.8% | 31.3 [17.89-61.03] | <0.001 | 40.7% | 34.7 | <0.001 |

OR, odds ratio.

**Supplementary Table S2** Adjusted mortality rate in relation to age and period, adjusted for male sex

| Age-group | Adjusted mortality rate, Wildtype | Adjusted mortality rate, Alpha | Adjusted mortality rate, Delta | Adjusted mortality rate, Omicron | Wildtype vs. Omicron | |
| --- | --- | --- | --- | --- | --- | --- |
|  |  |  |  |  | **OR** | p-value |
| 26–35 years | 2% | 1.5% | 1.1% | 0.5% | 3.7 [2.5-5.5] | 0.001 |
| 36–45 years | 2.6% | 1.9% | 1.5% | 0.7% | 3.7 [2.5-5.5] | <0.001 |
| 46–55 years | 7% | 5.4% | 4.1% | 2% | 3.7 [2.5-5.5] | <0.001 |
| 56–65 years | 12% | 9.2% | 7.1% | 3.5% | 3.7 [2.5-5.5] | <0.001 |
| 66–75 years | 18.9% | 14.8% | 11.5% | 5.9% | 3.7 [2.5-5.5] | <0.001 |
| 76–85 years | 28.3% | 22.8% | 18.1% | 9.6% | 3.7 [2.5-5.5] | <0.001 |
| >85 years | 41.4% | 34.6% | 28.4% | 16.0% | 3.7 [2.5-5.5] | <0.001 |

OR, odds ratio

**Supplementary Table S3** Observed mortality rate and adjusted mortality rate in relation to sex, adjusted for age (66–75 years)

| Sex | Total  (N=11,765) | No Death  (N=10,224) | Death  (N=1,541) | Mortality rate, observed | Comparison of male vs. female, observed | | Adjusted mortality rate, adjusted | Comparison of male vs. female, adjusted | |
| --- | --- | --- | --- | --- | --- | --- | --- | --- | --- |
|  |  |  |  |  | OR | p-value |  | OR | p-value |
| Male | 6,687 (56.8%) | 5,676 (55.5%) | 1,011 (65.6%) | 15.1% | 0.65 | <0.001 | 18.4% | 0.48 | <0.001 |
| Female | 5,078 (43.2%) | 4,548 (44.5%) | 530 (34.4%) | 10.4% |  | | 10.6% |  | |

OR, odds ratio.

**Supplementary Table** S4 Adjusted mortality rate in relation to SARS-CoC-2 period and sex, adjusted for age (66–75 years)

| Sex | Adjusted mortality rate, Wildtype | Adjusted mortality rate, Alpha | Adjusted mortality rate, Delta | Adjusted mortality rate, Omicron | OR, [CI], Wildtype versus Omicron |
| --- | --- | --- | --- | --- | --- |
| Male | 18.9% | 14.8% | 11.5% | 5.9% | 3.7 [2.49-5.51] |
| Female | 10.1% | 9.6% | 10.2% | 5.3% | 1.8 [1.03 -2.98] |

OR, odds ratio.

**Supplementary Table S5** Comparison of adjusted mortality rate between the Wildtype and Omicron periods in patients with different numbers of comorbidities

| No. of comorbidities | OR, [CI] | p-value |
| --- | --- | --- |
| 0 | 1.22 [0.56-2.65] | 0.61 |
| 1 | 2.77 [1.53-5.03] | <0.001 |
| 2 | 6.58 [2.97-14.61] | <0.001 |
| 3 | 3.76 [1.81-7.78] | <0.001 |
| >=4 | 6.47 [2.3-18.2] | <0.001 |

No., number; OR, odds ratio.

**Supplementary Table** S6 Comparison of adjusted mortality rate between patients with 0 versus ≥4 comorbidities within SARS-CoV-2 periods

| No. of comorbidities compared → / within period ↓ | ≥4 vs.0 | |
| --- | --- | --- |
|  | **OR, [CI]** | **p-value** |
| Wildtype | 2.88 [2.16-3.85] | <0.001 |
| Alpha | 3.37 [1.64-6.94] | <0.001 |
| Delta | 0.85 [0.34-2.19] | 0.979 |
| Omicron | 0.59 [0.17-1.93] | 0.424 |

**Supplementary Table S7** Comparison of adjusted mortality rate between the Wildtype and Omicron periods in patients with different comorbidities

| Comorbidity-group | OR [CI] | p-value |
| --- | --- | --- |
| Cardiovascular disease | 3.36 [1.78 - 6.16] | <0.001 |
| Pulmonary disease | 1.09 [0.46-2.63] | 0.834 |
| Renal disease | 1.38 [0.47-3.30] | 0.523 |
| Liver disease | 0.87 [0.20-3.74] | 0.893 |
| Oncological disease | 2.02 [0.72-5.64] | 0.141 |
| Severe immunosuppression | 7.07 [2.32-21.5] | <0.001 |
| Diabetes | 2.14 [0.87-5.31] | 0.139 |
| No comorbidities | 1.49 [0.76-1.85] | 0.182 |

OR, odds ratio.

**Supplementary Table S8** Univariate analysis of mortality rate in patients with different comorbidities independent of vaccination status

| Variable | Variable present | No Death  (N=10,224) | Death  (N=1,541) | Mortality rate | OR [CI] | p-value |
| --- | --- | --- | --- | --- | --- | --- |
| Vaccinated | No | 4,265 (41.7%) | 700 (45.4%) | 14.1% | 1.8 [1.4-2.3] | <0.001 |
|  | Yes | 851 (8.3%) | 78 (5.1%) | 8.4% |  |  |
|  | Missing | 5,108 (50.0%) | 763 (49.5%) | 13.0% |  |  |
| Cardiovascular disease | No | 4,715 (46.1%) | 389 (25.2%) | 7.6% |  | <0.001 |
|  | Yes | 5,440 (53.2%) | 1,140 (74.0%) | 17.3% | 2.5 [2.3-3.1] |  |
|  | Missing | 69 (0.7%) | 12 (0.8%) | 14.8% |  |  |
| Pulmonary disease | No | 8,600 (84.1%) | 1,168 (75.8%) | 12.0% |  | <0.001 |
|  | Yes | 1,388 (13.6%) | 314 (20.4%) | 18.4% | 1.7 [1.5-1.9] |  |
|  | Missing | 236 (2.3%) | 59 (3.8%) | 20.0% |  |  |
| Renal disease | No | 8,071 (78.9%) | 930 (60.4%) | 10.3% |  | <0.001 |
|  | Yes | 1,682 (16.5%) | 528 (34.3%) | 23.9% | 2.7 [2.4-3.1] |  |
|  |  | 471 (4.6%) | 83 (5.4%) | 15.0% |  |  |
| Liver disease | No | 9,686 (94.7%) | 1,425 (92.5%) | 12.8% |  | 0.55 |
|  | Yes | 271 (2.7%) | 44 (2.9%) | 14.0% | 1.1 [0.8-1.5] |  |
|  |  | 267 (2.6%) | 72 (4.7%) | 21.2% |  |  |
| Severe immunosuppression | No | 6,888 (67.4%) | 951 (61.7%) | 12.1% |  | 0.99 |
|  | Yes | 1,479 (14.5%) | 204 (13.2%) | 12.1% | 1.0 [0.9-2.1] |  |
|  | Missing | 1,857 (18.2%) | 386 (25.0%) | 17.2% |  |  |
| Oncological disease | No | 9,030 (77.5%) | 1,288 (83.6%) | 12.5% |  | <0.001 |
|  | Yes | 944 (9.2%) | 188 (12.2%) | 16.6% | 1.4 [1.2-1.7] |  |
|  | Missing | 250 (2.4%) | 65 (4.2%) | 20.6% |  |  |
| Diabetes | No | 7,923 (77.5%) | 1004 (65.2%) | 11.2% |  | <0.001 |
|  | Yes | 2,117 (20.7%) | 498 (32.3%) | 18.3% | 1.9 [1.7-2.1] |  |
|  | Missing | 184 (1.8%) | 39 (2.5%) | 17.7% |  |  |

OR, odds ratio.

**Supplementary table S9** Distribution of vaccination status in relation to SARS-CoV-2 period

| Vaccination status | SARS-CoV-2 period | | | |
| --- | --- | --- | --- | --- |
|  | Wildtype (N=8,800), total, (%), | Alpha (N=1,277), total, (%) | Delta (N=897), total, (%) | Omicron (N=791), total, (%) |
| Vaccinated (Total)  1x vaccinated  2x vaccinated  3x vaccinated  4x vaccinated | 32 (0.4%)  32 (100%) | 79 (6.2%)  55 (69.6%)  23 (29.1%)  1 (1.3%) | 363 (40.5%)  57 (15.7%)  288 (79.3%)  18 (49.6%) | 455 (57.5%)  23 (5.05%)  157 (34.5%)  221 (48.6%)  54 (11.9%) |
| Not vaccinated | 3,513 (39.9%) | 940 (73.6%) | 412 (45.9%) | 100 (12.6%) |
| Missing data | 5,255 (59.7%) | 258 (20.2%) | 122 (13.6%) | 236 (29.8%) |

**Supplementary Table S10** Adjusted mortality rate in relation to SARS-CoV-2 period and vaccination status, adjusted for age and sex

| Vaccine status | Adjusted mortality rate in relation to SARS-CoV-2 period | | | | | | | | | | |
| --- | --- | --- | --- | --- | --- | --- | --- | --- | --- | --- | --- |
|  | **Wildtype** | | **Alpha** | | | | **Delta** | | | **Omicron** | |
| Unvaccinated | 18.9% | | 14.6% | | | | 13.4% | | | 12.5% | |
| Vaccinated | 15.2% | | 16.2% | | | | 10.1% | | | 4.5% | |
| Comparison of adjusted mortality rate between unvaccinated and vaccinated patients | OR [CI] | p-value | | OR [CI] | p-value | OR [CI] | | p-value | OR [CI] | | p-value |
|  | 1.4 [0.7-3.6] | 0.7 | | 0.9 [0.7-1.3] | 0.129 | 1.4 [1.1-1.7] | | 0.078 | 3.0 [2.6-4.1] | | <0.001 |

OR, odds ratio.

**Supplementary Table S11** Distribution of antiviral therapy in severely immunosuppressed patients

|  | Wildtype, antiviral therapy/ total (%) | Alpha, antiviral therapy/ total (%) | Delta, antiviral therapy/ total (%) | Omicron, antiviral therapy/ total (%) |
| --- | --- | --- | --- | --- |
| Severely immunosuppressed | 137/1,149 (11.9%) | 30/152 (19.7%) | 53/130 (40.8%) | 152/252 (60.3%) |
| Not severely immunosuppressed | 940/5,486 (17.1%) | 191/1,083 (17.6%) | 153/748 (20.5%) | 109/522 (20.9%) |
| Missing data | 72/2,165 (3.3%) | 1/42 (2.4%) | 0 | 0 |

Supplementary figures:


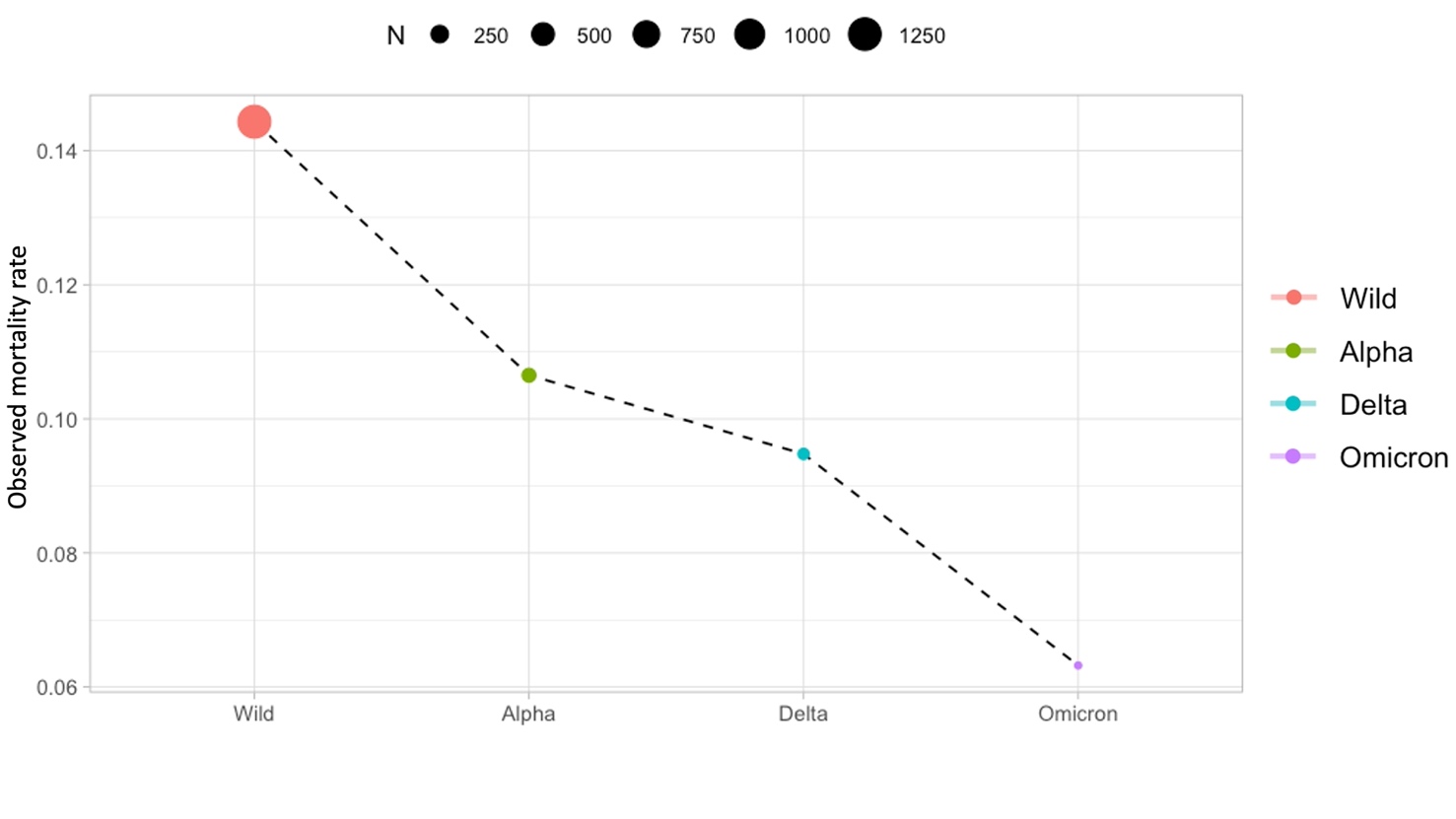


**Supplementary Figure S1** Observed mortality rate in relation to SARS-CoV-2 period

N, number.

**
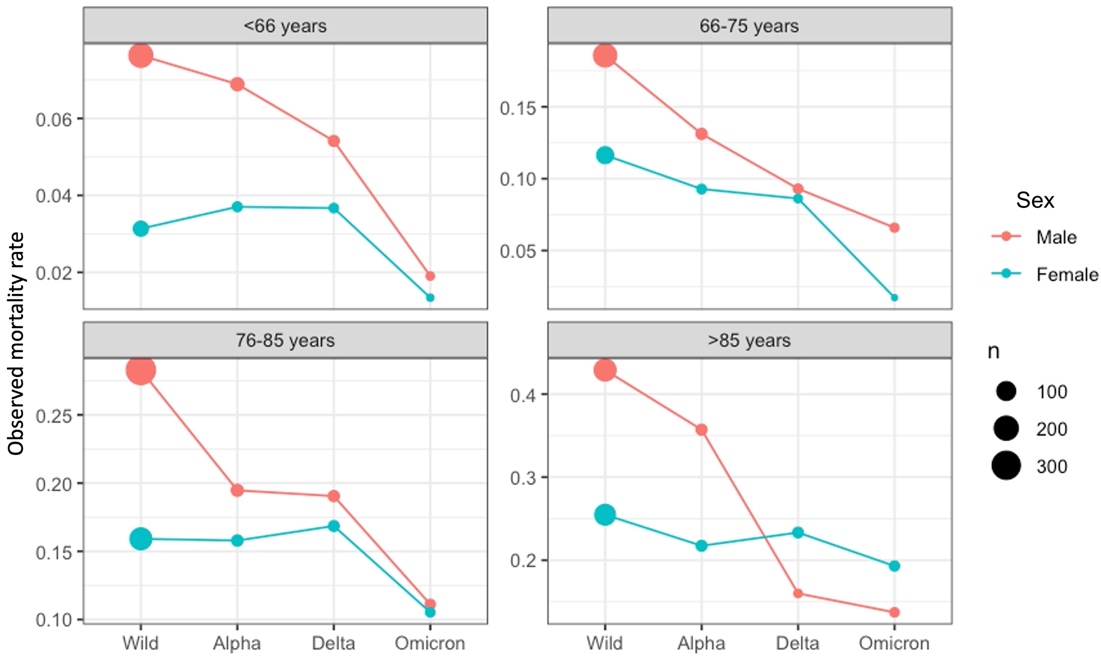
**

**Supplemetary Figure S2** Observed mortality rate in relation to SARS-CoV-2 period and sex in different age groups

**
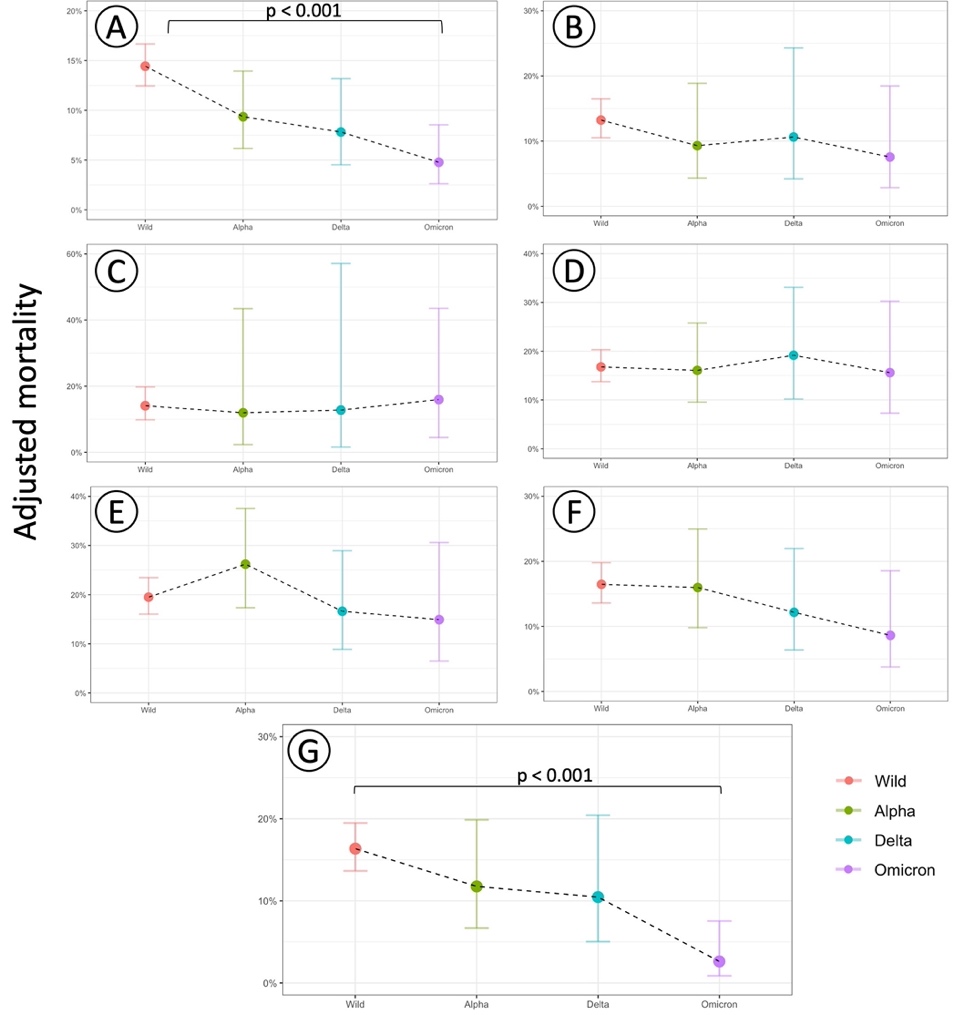
**

**Supplementary Figure S3** Adjusted mortality rate in relation to SARS-CoV-2 period and comorbidity

**A)** Cardiovascular disease, **B)** Oncological disease, **C)** Liver disease, **D)** Pulmonary disease, **E)** Renal disease, **F)** Diabetes, **G)** Severe immunosuppression.

**
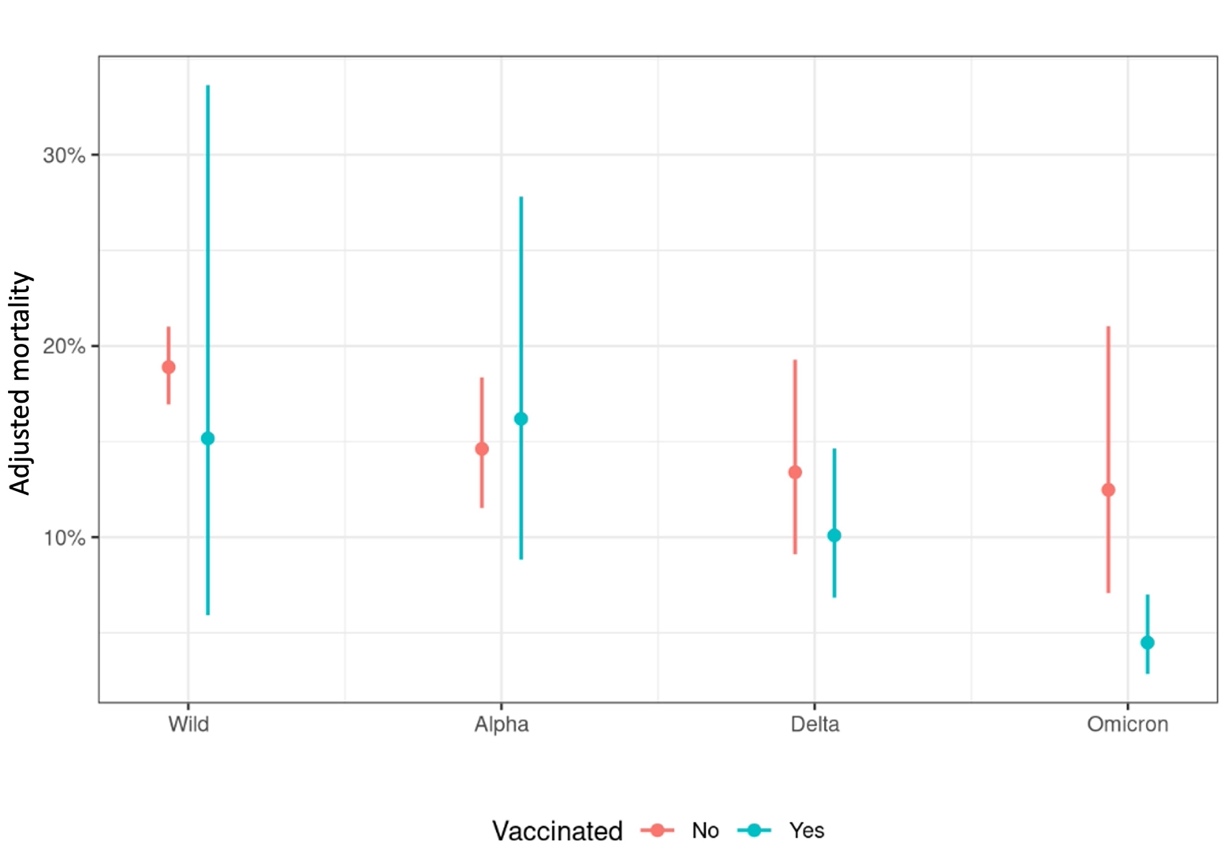
**

**Supplementary Figure S4** Adjusted mortality rate in relation to SARS-CoV-2 period and vaccination status


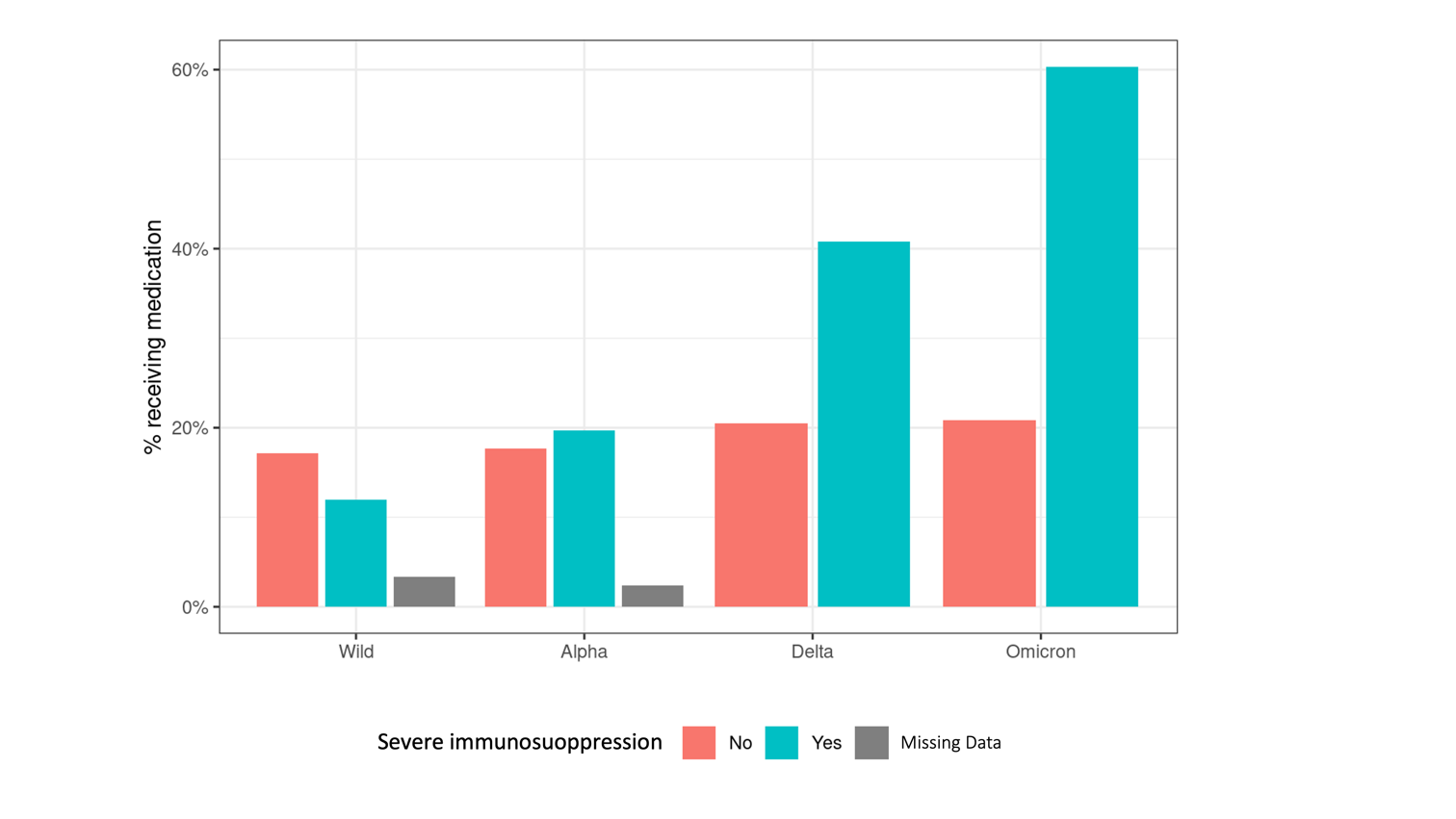


**Supplementary Figure S5** Use of antiviral agents across all SARS-CoV-2 periods in relation to immunity status
